# Foreign Patients Visiting the Emergency Department: A Systematic Review of Studies in Japan

**DOI:** 10.31662/jmaj.2022-0177

**Published:** 2023-03-13

**Authors:** Soichiro Saeki, Yohei Kurosawa, Koichiro Tomiyama, Rie Tomizawa, Chika Honda, Kaori Minamitani

**Affiliations:** 1 Department of Emergency Medicine and Critical Care, Center Hospital of the National Center for Global Health and Medicine, Tokyo, Japan; 2 Department of Nursing, Graduate School of Nursing, Osaka Metropolitan University, Osaka, Japan; 3 Department of Public Health Nursing, Shiga University of Medical Science, Shiga, Japan; 4 Department of International Medical Care, Rinku General Medical Center, Osaka, Japan; 5 Department of Public Health, Graduate School of Medicine, Osaka University, Osaka, Japan

**Keywords:** migrant health, minority health, global health, non-national patients, foreigners

## Abstract

**Background:**

As the number of non-native patients in Japan is increasing, emergency departments must provide proper care for international patients. However, no research has been conducted to determine the demographics of international patients that visit Japanese hospitals or the requirements to accept them. We aimed to organize the existing research and its patterns for foreign patients in Japan’s emergency departments and to identify the areas that require further research.

**Methods:**

Systematic review of research articles indexed in MEDLINE and Ichushi-web (Japanese medical literature) was conducted. The search strategy was based on a previous study in Japanese, and the search was limited to manuscripts published from 2015.

**Results:**

Nine publications that reported on the demographic characteristics of foreign patients who visited the emergency department were among the study’s 13 references. Injury diagnoses and the Asian population were both common. Dealing with overseas patients can be challenging due to linguistic barriers, cultural differences, and payment issues. However, studies describing the spoken language and the type of healthcare insurance used were lacking. Furthermore, neither the definition of “foreign patients” nor the distinction between short-term visitors and long-term residents were made in the majority of the research.

**Conclusions:**

The demographic characteristics of patients differed depending on the location and facility, despite the fact that several characteristics of foreign patients in emergency departments appeared to be generalizable. The COVID-19 pandemic may modify the demographic characteristics of immigrants; thus, more research from a broad range of locations and medical facilities is still necessary.

## Introduction

Migrant health is increasingly a worldwide issue in global health, even in high-income countries such as Japan ^(1)^. This covers the medical care for new immigrants or those who are descended from them, who may have varying histories from those of other local patients. The number of international tourists visiting Japan has climbed around six times since the start of the twenty-first century, despite a sharp decline in 2020 ^(2)^. In terms of foreign nationals living in Japan, 2.30% of the country’s population is made up of non-Japanese residents ^(2)^.

However, until recently, when the Japanese government started to implement measures to deal with the rising number of international tourists, the healthcare of patients without a Japanese identity was underappreciated ^(3)^. This includes developing practical guidance for patients with limited Japanese proficiency (LJP) ^(4)^ and accrediting certain medical facilities competent of delivering healthcare to foreign patients ^(5)^. Such measures were accelerated as Tokyo began investing in local prefectural governments to prepare for the Tokyo Olympic Games ^(3)^.

Nonetheless, many Japanese hospitals remain unprepared to deal with foreign patients. According to a national survey conducted by the Japan Hospital Association, approximately 95% of the hospitals in Japan answered that they were worried of linguistic issues if they were to deal with foreign patients ^(6)^. Previous studies on emergency departments in Japan have also highlighted the lack of linguistic assistance as a barrier in treating foreign patients ^(7)^, but studies to unravel further implications in the care for foreign patients have been lacking ^(8)^. This has been highlighted by the systematic review conducted by Tatsumi et al. ^(8)^ in 2016, when they found very few nationwide studies, as well as longitudinal or intervention studies. Furthermore, they did not find any studies in Japan that reported the characteristics of short-term foreign visitors ^(8)^.

To date, no study has been conducted to update the findings of Tatsumi et al. ^(8)^ As Japan has been eager to expand its ability to accept foreign patients, we projected that more research have been conducted in Japan in line with the growth in the trend to organize the environment for foreign patients.

Furthermore, the demographic features of foreign patients in Japan remain unknown. Identifying demographic features of foreign patients is important, as the medical requirements of foreign patients can be completely different from the locals ^(9), (10), (11), (12)^. Previous epidemiological studies have identified disparities between race and ethnicity in both acute ^(13), (14), (15)^ and chronic ^(16), (17), (18)^ medical conditions. Moreover, medical care for foreign patients requires detailed care focused on their linguistic, cultural, and religious backgrounds ^(19), (20), (21)^. Hence, understanding the current demographic features and the medical needs of foreign patients is of great necessity from the perspectives of public health and emergency medicine. Therefore, this study aims to identify major trends in Japanese studies to identify the characteristics of foreign patients in Japan and provide its key findings, as well as to indicate the research methodologies needed for studies that target foreign patients.

## Materials and Methods

We conducted a systematic review focused on foreign patients in the emergency departments located in Japan. The review was organized according to the PRISMA statement ^(22)^.

### Search strategy

Two databases, MEDLINE and Ichushi-web (Japanese medical literature), were searched on June 26, 2022. The search included keywords such as “foreigner,” “foreign patient,” “Japan,” and “emergency department” in English (MEDLINE) and Japanese (Ichushi-web). The search was limited to English and Japanese language manuscripts, published from January 1, 2015, to June 25, 2022, to cover for new literature that were published after the previous review ^(8)^.

### Eligibility criteria

All study designs that were (1) focused on foreign patients, (2) conducted in or reported from Japan, and (3) in the settings of pre-hospital emergencies or in the emergency departments were included for this study review. As the information to identify demographic features of foreign patients in emergency departments were focused on this study, case reports were excluded from the study.

### Study selection and data extraction

Two authors independently reviewed every title and abstract of the identified manuscripts for inclusion and exclusion based on the eligibility criteria, completely blinded by each of the authors by using the Rayyan platform ^(23)^. The full-text papers were read to confirm the final inclusion decision. Disagreements were resolved upon discussion of the authors.

From the full-text articles presented, one author independently retrieved the data to report for this study. The following information was extracted from each study: publication year, study duration, study type, location of the study, study population, nationality of the patients, outcome, key findings, and how “foreign patients” were defined in each study. For the studies that reported the demographic features of the patients, the method of arrival, arrival time to the emergency department, nationality of the patients according to regions, diagnosis, and information regarding to the payment including unpaid medical expenses were obtained. No meta-analysis was conducted due to the nature of this study.

## Results

### Study inclusion

**Figure 1.**
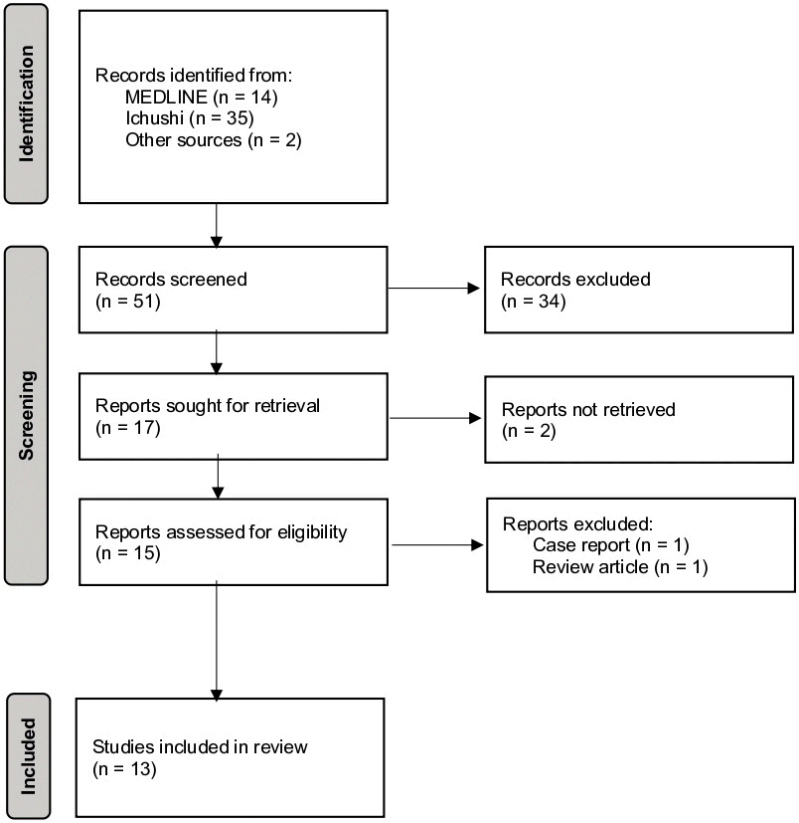
Inclusion and exclusion criteria of studies included in the study. The study was conducted following the PRISMA statement.

Figure 1 shows the inclusion and exclusion process according to the PRISMA statement. Forty-nine citations were found after searching the database, and two relevant citations not included in the databases were included. Upon initial title and abstract screening, 17 papers satisfied the inclusion criteria. Following a full-text review, two studies were excluded because one was categorized as a research article, while its content turned out to be a case report without study implications, and the other was the review by Tatsumi et al. ^(8)^ Thirteen manuscripts were evaluated qualitatively.

### Description of the included studies

Of the 13 studies identified, 9 ^(24), (25), (26), (27), (28), (29), (30), (31), (32)^ were reported from hospitals reporting the demographic characteristics of foreign patients visiting the emergency department. Three studies were longitudinal studies ^(33), (34), (35)^ and one study was a quantitative study conducted targeting foreign patients in the emergency department ^(36)^. Regarding the years of publication, 2020 saw the most publications with four, followed by 2019 with three and 2018 and 2022 with two publications each. 2016 and 2017 had one publication each, and no studies were included in our review from 2021.

### Demographic characteristics of foreign patients visiting Japanese emergency departments

Table 1 shows the overview of the nine studies with the demographic characteristics of foreign patients visiting the emergency departments of Japan. All studies were conducted through a retrospective review of healthcare records, while one study reported results of the analysis from payment records also. The location of the hospitals of which the study was conducted ranged from the north to Hokkaido and Okinawa on the south. Two studies particularly focused on short-term visitors, but no study provided the specific information on how they defined their population or how they have been able to classify the patients as foreigners (Table 1-a).

**Table 1-a.** Overview of the Studies Describing Demographic Features of the Patients in Emergency Departments of Each Facility.

	Author	Year	Study duration	Study type	Location	Definition of “foreigners”	Population
24	Takashina et al.	2017	January 2011―December 2015	Retrospective review of medical records and accounting data	Kyoto	definition of “visitors” Provided	“Visitors” that visited or were referred to the emergency department
25	Taguchi et al	2018	April 2012―March 2016	Retrospective review of medical records	Hokkaido	Not provided	Foreign patients
26	Oshita et al	2019	April 2015―March 2018	Retrospective review of medical records	Yamanashi	Not provided	Foreign visitors
27	Kainuma et al	2019	April 2014―March 2019	Retrospective review of medical records	Osaka	Not provided	Foreign patients
28	Suzaki et al.	2019	April―September 2017	Retrospective review of medical records	Tokyo	Not provided	Foreign patients visiting the primary and secondary emergency department for the first time
29	Nakazawa et al.	2020	January 2014―December 2018	Retrospective review of medical records	Okinawa	Not provided	Pregnant foreign patients in a single emergency department
30	Shimoyama et al.	2020	April 2015―March 2018	Retrospective review of medical records	Tokyo	Not provided	Foreign patients transported to a tertiary care center
31	Aoki et al.	2022	April 2018―March 2020	Retrospective review of medical records	Nagano	Not provided	Foreign patients transported to a tertiary care center
32	Ishii et al.	2022	January 2010―December 2019	Retrospective review of medical records	Tokyo	Visual classification of patient names	Foreign patients transported to a tertiary care center

The year of publication, the duration when the study was conducted, each facility’s location (in prefecture), how each study defined foreigners, and the study population was derived from each study.

All studies reported most of the patients being able to be discharged without requiring hospitalizations. However, the rate of patients arriving using ambulances or by walk-ins varied between studies. The arrival time also varied between each study (Table 1-b).

**Table 1-b.** Outcome, Severity, and the Time of Visit of Foreign Patients Defined in Each Study.

	Author	Year	Outcome			Transportation			Visits		
			Total patients								
			(included in the study)	Hospitalized	Deaths	Walk-in	Ambulance	Air transport	During daytime	During evenings	During nights
24	Takashina et al.	2017	1059	46 (4%)	1 (0%)	744 (70%)	314 (30%)	0	-	-	-
25	Taguchi et al	2018	132	-	-	-	132 (100%)	-	53 (40%)	49 (37%)	30 (33%)
26	Oshita et al	2019	474	-	1 (0%)	380 (80%)	93 (20%)	1	205 (43%)	269 (57%)	-
27	Kainuma et al	2019	198	13 (7%)	3 (2%)	56 (28%)	142 (72%)	0	142 (71%)	56 (29%)	
28	Suzaki et al.	2019	158	3 (2%)	0	131 (83%)	27 (17%)	0	110 (70%)	48 (30%)	
29	Nakazawa et al.	2020	37	4 (11%)	0	-	-	-	15 (41%)	11 (30%)	11 (30%)
30	Shimoyama et al.	2020	87	55 (63%)	25 (29%)	-	-	-	-	-	-
31	Aoki et al.	2022	777	71 (9%)	1 (0%)	660 (85%)	106 (14%)	11 (1.4%)	405 (52%)	262 (34%)	110 (14.2%)
32	Ishii et al.	2022	325	-	27 (8.3%)	-	-	-	-	-	-

Hyphens are indicated where information was unretrievable. The percentages were calculated by the authors based on the number of total patients included in the study. Numbers indicated between several columns indicate the numbers as the sum of the indicated sections.

Regarding the nationality of the patients, Asia was the most common population. However, the second largest population varied between the studies (Table 1-c). For most of the studies that did not focus on a single specific diagnosis, injury was the most common diagnosis for the study population. However, infectious and gastrointestinal diseases were also found to be common (Table 1-d).

**Table 1-c.** The Nationality of the Patients Reported in Each Study.

	Author	Year	Nationality						
			Asia	Europe	Oceania	North America	Latin America	Others	Unknown
24	Takashina et al.	2017	477 (45%)	201 (19%)	85 (8%)	148 (14%)	0	169	0
25	Taguchi et al	2018	84 (64%)	3 (2%)	-	-	-	0	25
26	Oshita et al	2019	369 (78%)	42 (9%)	24 (5%)	34 (7%)	1 (2%)	0	4
27	Kainuma et al	2019	157 (79%)	23 (12%)	11 (6%)	6 (3%)	0	1	0
28	Suzaki et al.	2019	123 (78%)	8 (5%)	0	12 (8%)	0	15	0
29	Nakazawa et al.	2020	32 (86%)	0	2 (5%)	2 (3%)	1 (3%)	0	0
30	Shimoyama et al.	2020	51 (74%)	6 (9%)	4 (6%)	5 (7%)	0	7	18
31	Aoki et al.	2022	-	-	-	-	-	-	-
32	Ishii et al.	2022	-	-	-	-	-	-	-

Hyphens are indicated where information was unretrievable. The percentages were calculated by the authors based on the number of total patients included in the study.

**Table 1-d.** Diagnosis of Diseases Foreign Patients Presented within the Emergency Departments.

	Author	Year	Disease											
												Infectious		Others/
			Injury	Urology	OBGYN	Pediatrics	Neurology	GI	Cardiovascular	Respiratory	ENT	diseases	Toxicology	unspecified
24	Takashina et al.	2017	295 (28%)	106 (10%)	21 (2%)	-	21 (2%)	164 (16%)	32 (3%)	202 (20%)	32 (3%)			169
25	Taguchi et al	2018	46 (35%)	5 (4%)	-	-	-	26 (20%)	-	11 (8%)	-	-	12 (9%)	32
26	Oshita et al	2019	166 (35%)	22 (5%)	21 (4%)	64 (14%)	168 (35%)	-	-	-	-	-	-	33
27	Kainuma et al	2019	72(36%)	5 (2%)	-	-	15(8%)	32(16%)	21(11%)	8(4%)	14 (7%)	-	-	31
28	Suzaki et al.	2019	19 (12%)	4 (3%)	0	0	4 (3%)	21 (13%)	2 (1%)	3 (2%)	3 (2%)	37 (23%)		92
29	Nakazawa et al.	2020	-	-	37 (100%)	-	-	-	-	-	-	-	-	-
30	Shimoyama et al.	2020	16 (18%)				4 (5%)		37 (31%)			3 (4%)	12 (14%)	24
31	Aoki et al.	2022	263 (33.9%)	47 (6.0%)	-	-	-	52 (67%)	-	114 (14.7%)	-	65 (8.4%)	-	56
32	Ishii et al.	2022	73 (21.8%)	0	-	-	14 (4.3%)	56 (17.2%)	73 (22.4%)	16 (4.9%)	-	17 (5.2%)	28 (8.6%)	48

Injuries include self-harms and burns. Cerebrovascular diseases are included in either cardiovascular or neurology, depending on the study. Cardiopulmonary arrests (CPAs) are included in cardiovascular diseases. Hyphens are indicated where information was unretrievable. The percentages were calculated by the authors based on the number of total patients included in the study. OBGYN, obstetrics and gynecology; GI, gastrointestinal; ENT, otolaryngology

Regarding the key findings in each study, linguistic issues posed as a major issue when dealing with foreign patients. In some hospitals, interpreting services were not commonly used, and communication difficulties were observed between the medical professionals and patients. Four studies mentioned about unpaid medical expenses by foreign patients (Table 1-e).

**Table 1-e.** Information on Payments by Foreign Patients and the Key Findings of Each Study.

#	Author	Year	Payment		Key findings
				Outstanding	
			Fee (JPY)	expenses	
24	Takashina et al.			68,000/outpatient,	
				745,000/inpatient,	Children accounted for 19.3% of the patients.
		2017	174500/day/person	1% of total	Many emergency transports were mild cases, and hospitalization was short at the average of 9.7 days.
25	Taguchi et al	2018	-	-	Cultural differences, linguistics, and religions posed as problems when dealing with foreign patients.
					Dealing with foreign patients requires a comprehensive approach with medical and non-medical staff.
26	Oshita et al	2019	-	-	There are differences in the needs of foreign patients between each location.
					Whether patients understand translational devices are unknown.
					Some required hospitalizations but refused; whether this is due to linguistic problems remains difficult to evaluate.
					Passport documentations and death certificates for other countries posed as difficulties.
27	Kainuma et al	2019	108,000 ± 453,340	6 (3%)	Communication was mainly possible with the usage of interpreting device.
					Unpaid medical expenses became a problem although many tourists had travel insurance.
28	Suzaki et al.	2019	-	3 (2%)	Infectious diseases, which were of common diseases, were also common among foreign patients.
					Primary and secondary emergency departments also had a high number of injuries.
					Some cases included several refusals of other medical facilities.
					Eight percent of patients with residency in Japan did not have a national healthcare insurance.
29	Nakazawa et al.	2020	-	-	Difficulty was found in retrieving information on the gynecological status of patients from other countries.
					Arrival in ambulances was common among foreign patients than Japanese patients.
30	Shimoyama et al.	2020	1,0009,735/case	13 (15%)	Forty-two percent of the patients were covered with a Japanese public healthcare insurance.
					Fifty-four percent of the cases required linguistic assistance, but professional interpreting was provided in only one case.
					Difficulty was found in gaining informed consent and planning international transfers.
31	Aoki et al.	2022	-	-	Language used when treating the patients was associated with length of stay in the emergency department.
32	Ishii et al.	2022	-	-	Anaphylaxis, burn, and infectious disease diagnoses were more common among non-Japanese patients.
					No statistical significance was found in mortality rates or mean lengths of stay with stratification on language or disorientation of the CNS.

Hyphens are indicated where information was unretrievable. The percentages were provided in each study. Key findings were derived quantitatively. JPY, Japanese yen

### Overview of quantitative and longitudinal studies

Table 2 summarizes the quantitative and longitudinal studies that focused on foreign patients who visited the emergency room of Japanese medical facilities. The three longitudinal studies were conducted in Osaka, while the qualitative study was conducted in a hospital in Tokyo.

**Table 2.** Overview of the Longitudinal and Qualitative Studies in the Review.

#	Author	Year	Duration	Study type	Location	Population	Definition of “foreigners”	Outcome	Key findings
33	Katayama et al	2016	Jan–Dec 2013	Retrospective study of EMS records	Osaka City	Patients using ambulances in Osaka City	Not defined	Characteristics of patients who experienced difficulty in hospital acceptance at the scene by emergency medical service personnel	Being a foreign patient was associated with difficulty in hospital acceptance at scene
									Difficulty may have occurred due to lack of multilingual staff in hospitals of Osaka
34	Kishi et al.	2018	Jan 10–July 31, 2018	Survey	Osaka Pref.	Hospitals with emergency departments in Osaka Prefecture	Not defined	Concerns when treating a foreign patient	Seventy-one percent of the facilities had difficulty as they had no interpreter. Thirty-eight percent of the hospitals experienced outstanding accounts.
35	Kishi et al.	2020	Jan 10–Dec 28, 2018	Survey	Osaka Pref.	Hospitals with emergency departments in Osaka Prefecture	Not defined	Concerns when treating a foreign patient	Seventy-two percent of the facilities had difficulty as they had no interpreter. Thirty-six percent of the hospitals experienced outstanding accounts. Issues regarding the patients’ perception toward medicine, religion, diet, and patient transfers arose as new problems in hospitals.
36	Beppu et al.	2020	2017.7–2018.1	Interview	Tokyo	Foreign patients over 20 years old that did not speak Japanese in an emergency department	Not provided	Pain, cultural difficulties, communication difficulties, worries on reoccurrence of the symptoms, and worries on payment were noted as difficulties.	Physical difficulties are more critical in emergency departments compared to the Japanese. Difficulties change during the clinical course.

The year of publication, the duration when the study was conducted, each facility’s location, how each study defined foreigners, the study population, outcomes, and key findings focused on foreign patients were derived from each study.

Of the longitudinal studies, one study was not necessarily focused on foreign patients. Rather, being a foreign patient was one of the key outcomes in the study mainly focused on the general population of Osaka City. The other two studies were surveys conducted among hospitals in Osaka Prefecture and highlighted the difficulties without interpreters and cases with outstanding accounts when dealing with foreign patients.

The qualitative study was an interview focused on the difficulties foreign patients face in the emergency department. This study also identified that the difficulties foreign patients experience change during over the clinical course.

## Discussion

In this review, we have summarized the studies conducted in Japanese emergency departments that focus on foreign patients. To date, our study is the first to review the studies that have been published near the Tokyo Olympic Games, which had been one of the key events that Japan focused on when organizing the environment to accept foreign patients ^(3)^.

Compared to the previous manuscript published in 2016 ^(8)^, three study trends arose. Firstly, the number of publications per year has increased. Secondly, reports that included information regarding short-term visitors were included. Thirdly, more studies reported the demographic features of foreign patients. These will all contribute to further understandings of non-national patients that visit the emergency department.

### Key trends from the results of the studies

Many studies focused on the demographic features of foreign patients. Many of the patients were from Asian countries, and many studies reported injuries to be common among foreign patients. As Japan is an island nation located in Asia, Asian patients being common is a reasonable result. Injuries are also reported to be common among short-term visitors in other countries ^(37)^, and this may be a trend that can be generalized for travel medicine. In addition, many foreign patients were discharged after receiving treatment.

Studies also highlighted the necessity for linguistic assistance. Linguistic assistance could aid communication to inform patients in detail about their health status ^(38), (39)^, and inadequate communication could have led to an excess amount of discharge for foreign patients. Furthermore, as foreign patients are more likely to experience financial and linguistic difficulties than local patients ^(40)^, a lack of effective communication may have increased the risk of outstanding accounts, when medical staff could not have provided information regarding the medical expenses necessary for treatment. As studies in this review have highlighted, providing linguistic assistance and financial support to indemnify for the outstanding accounts from foreign patients would be necessary when promoting Japanese hospitals to accept foreign patients in the emergency departments.

From the patient’s perspective, foreign patients are likely to experience unique difficulties when visiting Japanese emergency departments. As they receive treatment, they are likely to face cultural difficulties in addition to linguistic barriers, as well as to anxiety from the differences in the medical system compared to their homeland. Healthcare professionals should bear this in mind and try to communicate as effectively as possible using the greatest tools at their disposal, which may include medical interpreting services or other interpreting technologies.

### Implications for future studies

Although the number of studies increased during the recent years, further studies are still necessary to address the issues in migrant health in emergency departments. From this review, several factors that should be included in further studies, as well as future research questions, have emerged.

Firstly, criteria to report when discussing the demographic features of foreign patients must be clarified. One, how the study population were defined should be described more in detail. Although several studies have stated the definition of the “foreign patients” included in the study, no study reported the objective criteria that were used to define the patients. To fully define patients as foreigners, recording identifications may become necessary ^(12)^. Two, whether the patients were temporary visitors or residents should be disclosed. Some manuscripts reported demographic information of the patients only focused on visitors, but they did not state how they identified patients to be visitors. This would require recording the visa types or other documentations also ^(12), (41)^, which can also be troublesome for hospitals ^(42)^. Three, the type of insurance should also be reported. This would also aid grouping the patients as visitors or residents ^(12)^ and also generate a better idea on how to deal with outstanding expenses. Most hospitals keep record of the public healthcare insurance, and this data may be simpler to report than other official documentations. Four, the languages of the patients should be reported. Most studies reported linguistic difficulties, but the linguistics that was difficult to communicate with remains unclear.

Secondly, potential research questions arose from this study. One, studies that report the outcomes comparing the Japanese, foreign visitors and foreign residents would be necessary. It is thought that the medical demands of foreign visitors and residents are different ^(12)^, but the current studies have not been able to clearly identify such differences. Two, studies focused on linguistic communication would still be necessary. Compared to previous studies, more studies are reporting the use of linguistic assistance such as medical interpreting, but its advantages in the emergency departments in Japan remain unclear. Three, further studies reporting the demographics of overseas patients would be required. The novel coronavirus disease (COVID-19) pandemic has resulted in a dramatic decrease in the number of foreign visitors to Japan ^(2)^, and the demographic features of the patients may change. In addition, our study revealed that the demographic features of patients are different among regions, and thus, more studies from various regions would still be necessary.

### Limitations

This study has several limitations. Firstly, although we conducted the search for publications comprehensively including English and Japanese databases, publication bias is to be noted. We believe that many of the medical facilities in Japan that accept foreign patients do not routinely record these experiences, and the number of foreign patients in each facility may be few. The number of publications in this field may be decreased as a result of healthcare staff in such facilities failing to recognize their data as being important enough to publish. Secondly, the generalizability of this study should be addressed. Many of the facilities included in the study were accredited to accept foreign patients or had linguistic resources available. Data of such facilities may not be transferable to other facilities with fewer resources. Furthermore, longitudinal studies included in this manuscript were restricted to studies conducted in Osaka, which may not accurately represent the situations in other regions of Japan.

## Conclusion

This review provided an overview of the recent studies conducted in Japanese emergency department focused on foreign patients. As the number of studies increased until the COVID-19 pandemic, the number and variety of studies remain few. Further studies examining various features, especially the outcomes, would still be necessary. As the demographic features of foreign patients may change due to travel restrictions from during and after the COVID-19 pandemic, we await further studies to be conducted.

## Article Information

### Conflicts of Interest

None

### Author Contributions

Conceptualization, S.S.; methodology, S.S.; software, S.S.; validation, Y.K.; formal analysis, S.S.; investigation, S.S. and Y.K.; writing―original draft preparation, S.S.; writing―review and editing, Y.K., K.T., R.T., C.H., and K.M.; visualization, S.S.; supervision, K.T., R.T., C.H., and K.M. All authors have read and agreed to the published version of the manuscript.

### Approval by Institutional Review Board (IRB)

Not applicable
